# Seed Regeneration in *Taxus baccata*: Unveiling Ecological Restrictions and Paving the Way for Future Studies

**DOI:** 10.1002/ece3.70534

**Published:** 2024-11-11

**Authors:** Keyvan Maleki, Paweł Chmielarz, Mikołaj Krzysztof Wawrzyniak, Kourosh Maleki, Ahmad Maleki, Elias Soltani

**Affiliations:** ^1^ Department of Horticulture and Crop Science The Ohio State University Columbus Ohio USA; ^2^ Institute of Dendrology Polish Academy of Sciences Warsaw Poland; ^3^ Department of Forestry Gorgan University of Agricultural Sciences and Natural Resources Gorgan Iran; ^4^ Department of Agronomy and Plant Breeding Sciences, College of Aburaihan University of Tehran Tehran Iran

**Keywords:** English yew, morphophysiological dormancy, regeneration, seed predation, *Taxus baccata*

## Abstract

*Taxus baccata*, commonly known as English yew, is an evergreen tree native to regions ranging from Ireland and Sweden to Morocco, Algeria, and northern Iran. This species is of special concern due to habitat loss from human activities, including forest management, leading to declining populations. A 4‐year monitoring study was conducted to investigate the factors behind the poor seed regeneration of yew. We examined seed germination, dormancy, desiccation tolerance, and seed predation as potential contributors to this issue. Our study proposed potential seed predation by frugivores and granivores, along with morphophysiological dormancy, as primary reasons for poor regeneration. Despite high seed production and seed availability in certain years, germination did not improve, likely due to the small size of the yew seed embryos, which prolongs dormancy. Yew seeds are desiccation‐tolerant, water‐permeable, and lack physical germination barriers, making seed predation a significant limiting factor. In conclusion, the natural regeneration of yew is hampered by potential seed predation, morphophysiological dormancy, and environmental factors such as altered temperature and rainfall patterns, which change the dormancy‐breaking process. Further research is needed to quantify seed predation and explore its impact on seedling survival.

## Graphical abstract

In a comprehensive 4‐year study on *Taxus baccata*, we identified key factors hindering its seed‐based regeneration, notably high seed predation rates and morphophysiological dormancy. Despite the species' tolerance to desiccation and lack of physical germination barriers, seedling emergence remains low, suggesting that predation, along with environmental changes, critically limits natural regeneration. Our findings highlight the need for further research into seed and seedling predation dynamics to inform conservation strategies for this ecologically significant tree.

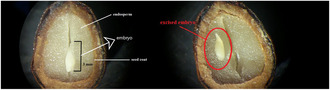

## 1 Introduction

With climate change projected to intensify, understanding the functioning of forest ecosystems has become essential for sustainability, particularly as these ecosystems rely heavily on seed‐based regeneration (Ruprecht et al. 2010; Soltani, Maleki, and Heshmati 2022). The English yew (*Taxus baccata* L.), an ancient conifer species, exemplifies this ecological challenge. Since the Middle Ages, *T. baccata* has suffered from climate fluctuations and anthropogenic pressures, with its decline in Europe signaling the onset of the contemporary biodiversity crisis (Malliarou et al. 2023). Studies underscore the crucial role of biodiversity in maintaining ecosystem functions and services, highlighting the necessity of conserving species like *T. baccata* (Jafari and Akhani 2008; Sabater‐Jara, Tudela, and López‐Pérez 2010; Sharma, Uniyal, and Slowik 2014). Despite intensified forest management efforts, the decline of *T. baccata* persists, with some practices exacerbating the species' endangerment and significantly reducing its populations (Malliarou et al. 2023; Moosavi et al. 2024). Given the ecological importance of *T. baccata* in forest ecosystems and its poor natural regeneration from seeds, this study aims to elucidate the ecological factors contributing to this regeneration deficit.

Seeds of *T. baccata*, as well as those of other species of Taxus, have a minute underdeveloped and physiologically dormant embryo, indicating deep simple morphophysiological dormancy. The initial length of the embryo in ripe seeds is 1.2–1.8 mm (LePage‐Degivry 1973; Devillez 1976), while the total length of the surrounding female gametophyte is 5–6 mm. Under natural conditions, seeds are dispersed in late summer and autumn, but they do not germinate until at least the second spring after dispersal (Wappes 1932; Anonim 1948; Heit 1969). Some seeds may even germinate in the following spring or later. The need for both warm and cold stratification for dormancy release, combined with the potential for increased average temperatures to compromise these thermal requirements, exacerbates this issue (Suszka 1985; Soltani, Maleki, and Heshmati 2022). Additionally, high levels of seed predation in the Hyrcanian forest further contribute to the poor regeneration of these species (Soltani, Maleki, and Heshmati 2022).

Another factor contributing to the poor regeneration of *T. baccata* from seed is that seeds only form where female trees coexist with male ones (Suszka 1985). Additionally, some authors report phenomena such as a lack of any regeneration or mass dying of young *T. baccata* seedlings in areas where this species is native or in some reserves (Król 1978). Higher germination percentages can be achieved in the second year and, more optimally, in the third year, although seed viability remains high for at least 4 years (Melzack 1979; Melzack and Watts 1982). According to Heit (1969), seeds of yew germinated up to 95% after a 3‐year interval, but this may not be reliable given the impact of climate change and seed predator pressure.

The regeneration of *T. baccata* from seed, a species of paramount ecological value, is hindered by its complex seed dormancy, which has been meticulously studied and addressed through various techniques (He et al. 2023). Suszka (1985) delineated a successful three‐phase treatment protocol, initiating with a 6.5‐month stratification at temperatures ranging from 15°C to 20°C, followed by a 4 to 4.5‐month period at 3°C, and culminating in a final germination phase at 20°C. This method has proven effective, with germination rates soaring to 80%–90% after the 10–11‐month pretreatment. Moreover, yew seeds exhibit remarkable longevity, remaining viable for up to 4 years in situ, with germination predominantly occurring in the second or third year postdispersal. Notably, the germination process is unaffected by light exposure and can even occur on bare rock if moisture is available, as Vogler (1904) observed, elucidating the species' adaptability to cliff environments across Europe. The findings of Melzack and Watts (1982) further reveal regional variations in seed size, correlating with the climatic conditions of their origin. The interplay of climatic factors—temperature, moisture, and the escalating threat of climate change—alongside biotic pressures such as seed predation, critically influences the fate of *T. baccata* seeds, underscoring the challenges faced in the conservation and regeneration of this ecologically important tree species.

Although recent studies have expanded on the challenges facing the regeneration of *T. baccata*, highlighting the potential of embryo rescue techniques to enhance germination success (He et al. 2023), the positive effects of shrub cover and soil moisture on seedling recruitment (Calvia et al. 2023), and the importance of genetic diversity for the species' adaptability and conservation (Malliarou et al. 2023), the ecological factors responsible for poor seed regeneration of *T. baccata* remain unclear. This study aims to address three critical questions: (1) whether yew produces an adequate quantity of seeds to support robust regeneration; (2) if there is a pattern of years with greater seed production, considering that years with greater seed production can reduce seed predation (Huang et al. 2021); (3) to what extent yew seeds are tolerant of desiccation, potentially affecting seed viability in natural settings; and (4) what type of seed dormancy this tree species exhibits and how it may affect seed regeneration. Although morphological dormancy in Taxaceae species is well‐documented (Baskin and Baskin 2014), the specific dormancy type in yew seeds remains unreported. By investigating these factors, this study seeks to elucidate the complex interplay between climatic and biotic factors that hinder the regeneration of yew. This comprehensive approach underscores the urgency of addressing the adverse impacts of climate change and seed predation on *T. baccata*, a species of paramount ecological value, to inform and improve conservation strategies and ensure the sustainability of forest ecosystems.

## 2 Material and Methods

### 2.1 Ecology, Distribution, and Conservation of *Taxus baccata*

The English or common yew (*Taxus baccata* L., Taxaceae) is an evergreen tree species endemic to Europe, northern Africa, and northern Iran (Rushforth 1999). It is a relict, diploid (2*n* = 24), wind‐pollinated gymnosperm (Gargiulo et al. 2019). The Hyrcanian region, representing the easternmost extent of *T. baccata*'s range, supports this species across a diverse altitudinal gradient, predominantly above 1000 m (Litkowiec, Lewandowski, and Wachowiak 2018). Research indicates that *T. baccata* can be found at elevations up to approximately 2100 m in this region (Karami‐Kordalivand et al. 2021; Hematzadeh et al. 2023). *T. baccata* has been categorized as an endangered tree species that is at risk of extinction due to the decreasing size and age of its populations (García et al. 2000). As a nonresinous gymnosperm, this species can grow up to 30 m in height, often with multiple trunks and a pyramidal canopy (Mossadegh 2002; Thomas and Polwart 2003). Despite being poisonous to mammals if ingested, *T. baccata* is widely grown as an ornamental (Handeland et al. 2017). In Europe, the distribution of this long‐lived species is extensive; however, its range has declined significantly across much of its habitat and is now confined to mountainous areas (Tittensor 1980; Hulme 1996). In Iran, the range of *T. baccata* is confined to the northern slopes of the Alborz Mountain, with sporadic occurrences in the Guilan and Mazandaran provinces. The region is characterized by a humid climate, especially on the northern slopes, which are fertile and support diverse vegetation types, including the Hyrcanian forests where *T. baccata* thrives (Habibi Kaseb and Lessani 1985; Noroozi, Talebi, and Doostmohammadi 2020). The southern slopes, in contrast, tend to be semiarid or arid. This diverse climatic gradient, influenced by elevation and proximity to the Caspian Sea, provides a variety of habitats that contribute to the ecological richness of the area (Noroozi, Talebi, and Doostmohammadi 2020).

Populations of *T. baccata* in the Polish part of the Carpathians occur in nature reserves, commercial forests, and private land. They occupy a total area of 721.2 ha, which is 65% of the total area of the species in the Polish part of the Carpathians. The distribution pattern of individuals in each of the studied populations is clustered, a rare phenomenon among native tree species that usually show a random distribution (Widlak 2022). *T. baccata* populations in the Polish part of the Carpathians exhibit a normal sexual structure and continuity of population development. A consequence of the clustered distribution of mature individuals, particularly fruit‐bearing females, is spatial variation in seed density (Widlak 2022).

In lowland Poland, *T. baccata* grows on diluvial sandy soils, silty‐sandy and clayey soils, and peat. The first protected area for *T. baccata* was established in Poland in the Tucholskie Forests in Gdańsk Pomerania in 1827. Currently, the species is protected in 33 nature reserves in Poland. There are *T. baccata* trees in four national parks. Moreover, in the Rokita Forestry Department in the northern part of Goleniowski Forest in the West Pomeranian Province, there are about 6000 trees outside the nature reserve; in the forests of the Dukla Forestry Department in the Beskids, more than 1300 trees; in private forests in the regions of Gorlice, Polnowa, and Szalowa in the Lesser Beskids, more than 1000 specimens; and in the forests of the Wipsowo Forestry Department in Masuria (southeast of Olsztyn), 800 trees. Spontaneous renewal of *T. baccata* has been observed in some regions of Poland, thanks to a successful method of seed preparation that results in a higher proportion of healthy seedlings. *T. baccata* is being reintroduced in forests as undergrowth in young pine and oak stands. Additionally, young stands are protected from being eaten by roe deer (Szeszycki 2006).

### 2.2 Seeds Collections

All experiments were conducted on collected seeds or trees from the Hyrcanian forests in Iran, except for the germination, seed viability, and seedling emergence trials of seeds collected in Poland. In Iran, seeds were collected from 10 trees in Ziarat, Gorgan (36°42′00.0″ N 54°36′00.0″ E; elevation: 2049 m; Figure S1) in 2016, and experiments were conducted on them. To collect seeds, we used a bulk collection method, which involved selecting robust plant populations to ensure genetic diversity and adaptability. We timed our collection to coincide with peak seed maturity. Postcollection, seeds were carefully processed to remove debris and nonviable seeds, then stored dry in an incubator (20°C, darkness, ambient humidity, 55%–60% relative humidity) prior to beginning the experiments. An experiment on seed desiccation tolerance was conducted using seeds collected in the Wierzchlas Reserve near Tuchola in the western part of Poland. All collected seeds were cleaned of arils, dried to a moisture content of 10%, and initially stored in tightly closed containers at a temperature of 3°C until the experiments began. Here, we aimed to test seeds at a wide range of moisture levels to see how desiccation tolerance may vary.

### 2.3 Seed Dormancy and Germination

#### 2.3.1 Seed Morphology

To gain a deeper understanding of the morphological characteristics of *T. baccata* seeds and their embryos, we used a well‐established method (Soltani, Maleki, and Heshmati 2022). Initially, the seeds were placed on a substrate imbued with moisture and incubated at a constant temperature of 20°C for a period of 16 h. This initial step was crucial to simulate early germination conditions, allowing for any physiological changes in the seed that might facilitate the subsequent examination. Following incubation, each seed was delicately sliced open along its longitudinal axis using a sterilized razor blade. This precise incision was performed to ensure minimal damage to the internal structures, particularly the embryo. The exposed seeds and embryos were then placed under a high‐resolution dissecting stereomicroscope equipped with a mounted micrometer, provided by Nikon.

Under the microscope, we meticulously measured the size, shape, and length of the embryos. These measurements were not only critical for understanding the physical attributes of the embryos but also for assessing their developmental stage. The dimensions were recorded with precision. With the collected data, we calculated the embryo‐to‐seed length ratio, a significant metric that reflects the embryo's development relative to the seed size. This ratio is indicative of the seed's maturity and potential germinability. A higher ratio suggests a well‐developed embryo, which could be a predictor of successful germination and seedling establishment, while a lower ratio indicates a morphological barrier that prevents seeds from germinating (Baskin and Baskin 2014).

#### 2.3.2 Germination and Seed Viability

The stratification treatment involved using a moist mixture of quartz glass sand (< 1 mm fraction) and sifted peat (pH 3.5–4.5) in a 1:1 volumetric ratio. Seeds were mixed with this substrate in a 1:3 ratio and placed in 0.25‐l plastic bottles. These bottles were sealed with lids containing 0.5 cm diameter holes to facilitate gas exchange. The conditions of the seeds and substrate were monitored weekly, with water added as necessary by spraying the stratification mixture. Seed viability, including fungal infections and insect larvae, was regularly checked. Early germination, indicated by seeds with a root size of 2–3 mm, was recorded to determine dormancy release. Each experimental variant consisted of four replicates, with 50 seeds per replicate. Stratification was carried out in a cyclically variable temperature regime of 15°C–20°C (24 + 24 h) for 6.5 months, followed by 3°C for 4.5 months, as per the method described by Suszka (1985).

Following completion of the germination test, nongerminated seeds underwent a final assessment, in which seed viability was determined by a cut test (4 × 50 seeds). Seeds were cut along the longitudinal axis using a scalpel. Among the nongerminating seeds, the following categories were distinguished: healthy, damaged, and empty seeds. Damaged seeds exhibited signs of decay due to primary infection (originating from their own seed). Such seeds did not develop. Empty seeds were classified as those containing < 50% seed tissue (Suszka 1985).

#### 2.3.3 Seedling Emergence Trial

To validate our findings during the stratification treatment and gain a better understanding of the germination ecology of yew, a seedling emergence experiment was performed. Seedlings were grown in the same sand and peat mixture used for stratification and germination tests. Stratified seeds were sown in plastic boxes, in the substrate at a depth of 1 cm, and covered with a layer of sand. To ensure appropriate moisture conditions, the boxes were covered with a light‐transparent lid. The lid was removed when the seedlings reached a height of about 2–3 cm. Seedling trials were conducted at 3°C–20°C (3°C at night and 20°C during the day) for 8 weeks, with a light intensity of 60 μmol m^−2^ s^−1^ (16 h/day). Each experimental variant included 4 replicates with 50 seeds each (Suszka 1985).

### 2.4 Seed Desiccation Experiments

To conduct research on the cryogenic storage of this species, permission was obtained for seed collection from the Regional Directorates for Environmental Protection. Seeds were collected in the “Cisowa Góra” reserve near Barda Śląska and in the “Cisy Staropolskie im. Leona Wyczółkowskiego” reserve in Wierzchleś near Tuchola. Seeds were collected from eight trees in Wierzchleś and one tree in the “Cisowa Góra” reserve. After collection, the seeds were cleaned of arils, dried to a moisture content of 12%, and initially stored in tightly closed containers at a temperature of 3°C. The seeds from eight trees in the “Cisy Staropolskie” reserve were mixed together; in the study, this is considered a single provenance—Wierzchlas. Nonstratified seeds were brought to 16 moisture levels and frozen in liquid nitrogen for 24 h at the same 16 moisture levels. The seeds were then placed in a stratification mixture at a temperature of 15°C/20°C (24 h/24 h). An analogous experiment on seed sensitivity to strong desiccation and liquid nitrogen temperature at the same 16 moisture levels (ranging from 1.6% to 32.1%) was conducted on seeds after dormancy release.

Nonstratified and stratified seeds were brought to 16 moisture levels, ranging from 1.6% to 31.1% and 1.7% to 32.1%, respectively. Depending on the targeted moisture level for the seeds, they were initially dried in the air, then over silica gel (stratified seeds) or moistened in a tightly closed container (seeds stored in a dried state), while monitoring the sample seed mass. The desired seed mass (X), corresponding to the intended moisture content (Wd), was calculated using the formula (Suszka 1978).

$$X=\frac{M\times \left(100-W_{p}\right)}{100-\mathrm{Wd}}$$

where 𝑋 is the mass to which the seeds needed to be moistened/dried to achieve the intended moisture content. 𝑀 denotes the fresh mass of moistened/dried seeds and 𝑊 𝑝 refers to the moisture content of the seeds before moistening/drying. 𝑊 𝑑 is the target moisture content of the seeds.

To achieve higher moisture levels (moistening), the seeds were sprayed several times with water until reaching a specified mass. Subsequently, they were left in tightly closed containers (conditioning) for a period of 2–3 days at a temperature of 3°C to equalize the moisture throughout the seed mass. Moisture levels below 12% were achieved by drying the seeds overactive (dried at 200°C) silica gel. Drying the seeds took several days, except for the lowest moisture level, which took 3 weeks. The seeds were placed on filter paper (in a layer not exceeding twice the height of the seed) in a box (25 × 15 × 9 cm) filled with 200 g of silica gel.

### 2.5 Seed Production and Predation

We selected 10 trees per area (plots of 0.1 ha per year) and monitored them over a 4‐year period to assess the impact of environmental factors and morphological features on the seed production of yew. To isolate the impact of climatic factors, we utilized the average temperature and precipitation data for each year of the study period (2016–2020). We attempted to understand how changes in climate may influence seed production by regressing the average number of seeds produced by 10 trees on the average temperature and precipitation of each year. This approach allowed us to assess the relationship between the average number of seeds produced and the climatic conditions, without the confounding effects of other environmental variables. By exploring the effects of temperature and precipitation on seed production, we aimed to enhance our understanding of the role of climatic factors in regulating seed production in plants.

The yew tree is found in remote areas of the Hyrcanian Forest, and there is a lack of information on the impact of predation on its regeneration. To address this knowledge gap, we conducted a 4‐year study of yew populations in the Hyrcanian Forest of Gorgan, Iran, and the forest of Ziarat, Gorgan (36°42′00.0″ N 54°36′00.0″ E) to determine the presence of seed predation, frugivory, and granivory. Our monitoring methods included the use of motion detection cameras and repeated field visits. For each tree, a seed trap was installed following the method described by Yang et al. (2015), and the monitoring was conducted annually from 2016 to 2020. A permanent 50 × 100 m monitoring plot was established for the 4‐year period, and within the plot, 10 seed traps were employed to track masting patterns and seed fate. The spacing between each seed trap was 7.1 m, nearly equal to the average diameter of the crown of mature *T. baccata* trees. A year of greater seed production was defined as a year when the mean seed density exceeded the standard deviation over the 4‐year period (LaMontagne and Boutin 2009). The seed traps were designed to enable animals to remove seeds to determine if predation is a cause for poor regeneration.

### 2.6 Data Analysis

Before analyzing the data, we checked for data normality by visually inspecting the histogram plots and Q‐Q plots of our dataset. Additionally, we performed statistical tests such as the Shapiro–Wilk test and the Kolmogorov–Smirnov test to quantitatively assess the distribution of the data. These preliminary steps ensured that the assumptions of our subsequent parametric statistical analyses were met. To see if seed production changes on a yearly basis, we used generalized linear model with binomial family function with Tukey adjustment to statistically compare the mean seed production across different years. This method allowed us to determine if there were any significant differences in seed output from year to year while controlling for the possibility of type I errors due to multiple comparisons. The Tukey post hoc test provided a pairwise comparison between each year, giving us a detailed understanding of which specific years differed significantly in terms of seed production. Germination capacity after desiccation of seeds to different moisture content was analyzed using linear regression. All the analysis were performed using R version 4.3.1.

## 3 Results

### 3.1 Seed Production

**FIGURE 1:**
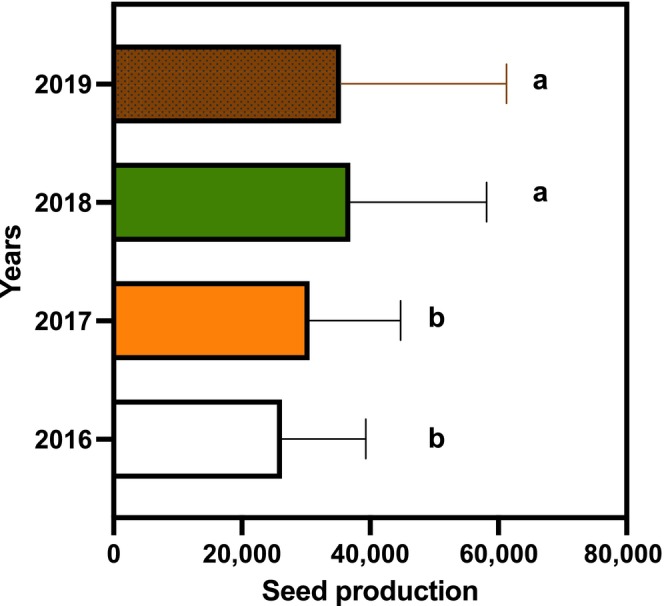
Seed production of yew in four consecutive years. A generalized linear model with binomial family function with Tukey adjustment to statistically compare the mean seed production across different years.

Years of high seed production are prevalent in the Hyrcanian forests. During the four consecutive years of the study, high seed production was observed in all years, with notable variations across the study period. The third and fourth years were particularly productive, with each tree yielding nearly 36,000 seeds on average (Figure 1). In contrast, the first and second years showed comparatively lower productivity, though the mean seed production value remained consistently higher than the standard deviation over the 4‐year period. This pattern suggests a trend of increasing seed production over time, which could be indicative of favorable environmental conditions or adaptive reproductive strategies in *T. baccata*.

### 3.2 Seed Production and Morphological Correlations

**FIGURE 2:**
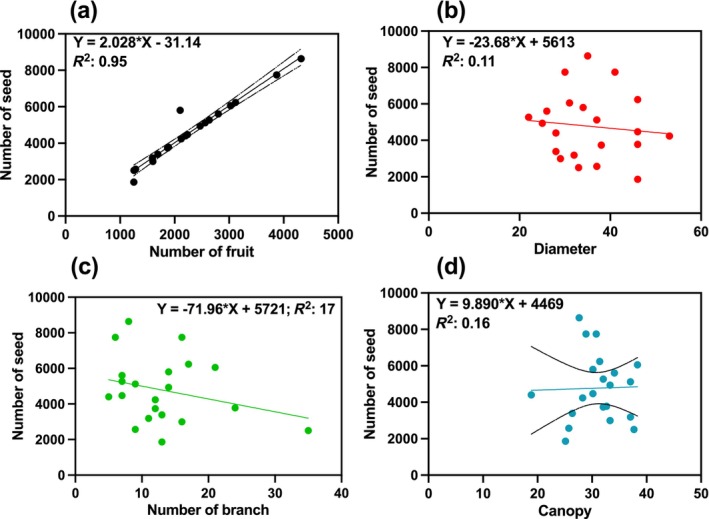
The relationship between seed production and morphological features. Number of seeds regressed on morphological features, including number of fruit (a), trunk diameter (b), number of branch (c), canopy width (d).

Our findings indicated a positive correlation between the number of fruits and canopy width (regression slopes of 2.02 and 9.89, respectively) and seed production (Figure 2a–d; Table 1S for further information). Conversely, other morphological features showed a negative correlation with seed production (Figure 4a–d).

**TABLE 1:** Seed germination and seedling emergence of yew as affected by stratification. The proportion of decayed and healthy seeds in the total number of seeds that did not germinate after stratification and germination capacity test. Maturity refers to harvest time.

Seed germination capacity	
Germination trait	Stratification
Germination percentage (%)	80 ± 2
Seedling emergence (%)	78 ± 1

Seed viability	
Viability status	Proportion of seeds (%)
Damaged seeds	25 ± 5
Healthy seeds	75 ± 3

### 3.3 Germination Status, Viability, and Desiccation Effect

**FIGURE 3:**
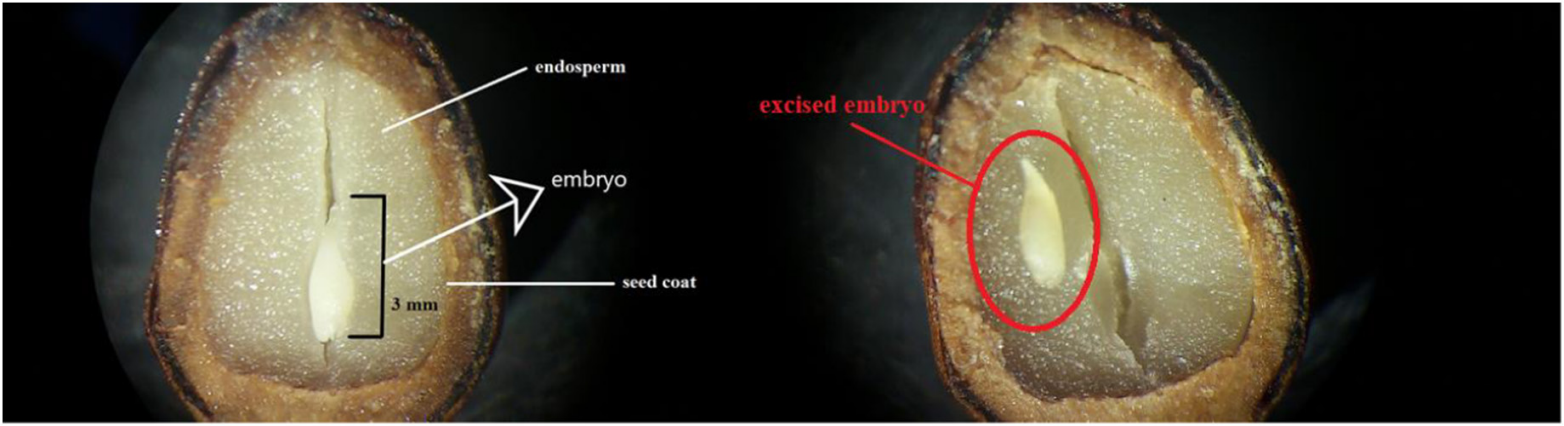
The photograph on the left shows longitudinal sections of *T. baccata* seeds, indicating small embryo relative to endosperm at the time of seed maturity and the right photographs indicates excised embryo of Yew.

Seeds of yew have a small and underdeveloped embryo, which took up less than 30% of the seed, as shown in Figure 3. On average (±SD), seed and embryo lengths were 10.1 ± 0.23 and 2.9 ± 0.03 mm, respectively, and the embryo‐to‐seed length ratio was 0.29 ± 0.004 (Figure 3). Furthermore, seeds of yew absorbed water readily without any issues, indicating that the seed coat is not a barrier to seed regeneration in yew (Figure S2).

**FIGURE 4:**
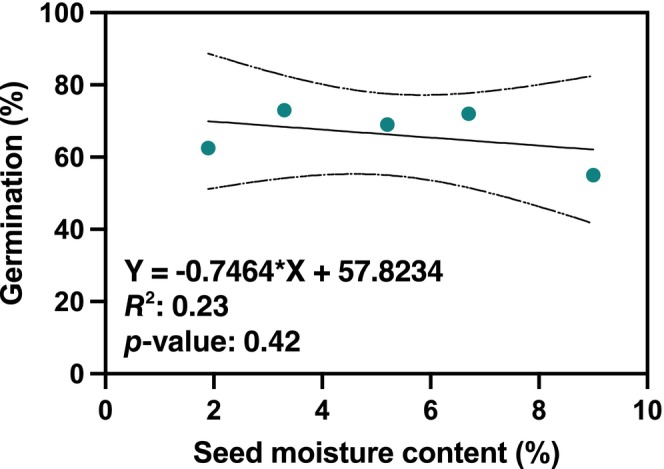
Effect of *Taxus baccata* seeds desiccation in the range 2%–9% on their germination after stratification. Collected seeds were desiccated from 9% to 1.9%, 3.3%, 5.2% and 6.7%. Provenance: Wierzchlas Reserve, Poland.

Seed dormancy was released following stratification, which improved the germination of yew seeds from zero at maturity to 80% (Table 1). Nearly 80% of seeds were viable (dormant), while only 20% were damaged and unhealthy (Table 1). Seed moisture content was negatively correlated with germination capacity, but this relationship was not statistically significant (slope: −0.746; *p* = 0.42; Figure 4).

### 3.4 Potential Seed Predation

**FIGURE 5:**
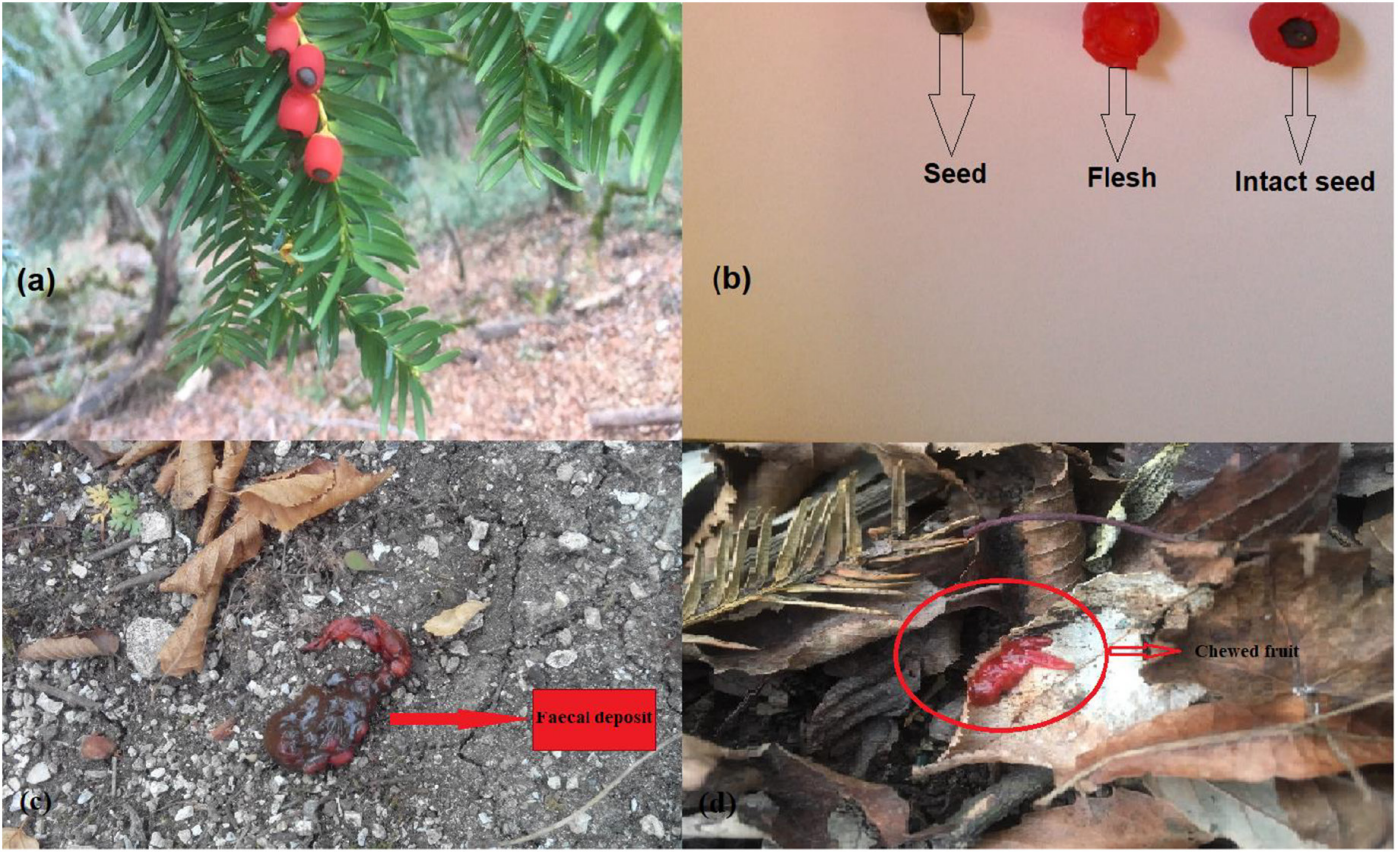
(a) Aril of yew attached to mother plant, (b) from the left, seed without aril, flesh and seed without aril, (c) the remains of seeds of yew in a fecal deposit of an animal, and (d) the remains of flesh chewed by an animal.

Although we did not obtain quantitative data identifying the specific animals or birds responsible for seed predation, our observational evidence suggests that yew seeds are likely attractive to various fauna due to their juicy and colorful flesh. This inference is supported by the discovery of partially eaten seeds in animal fecal deposits, indicating that animals play a role in the seed predation process (Figure 5).

## 4 Discussion

### 4.1 Seed Dormancy and Germination

In agreement with previous databases (Baskin and Baskin 2014 and references therein), seeds of *T. baccata* exhibit morphophysiological dormancy. Germination of these seeds after a warm stratification (15°C–20°C for 6.5 months) followed by a cold stratification (3°C for 4.5 months) improved germination rates up to 80%, indicating deep simple morphophysiological dormancy, as suggested by Baskin and Baskin (2014). However, Suszka (1985) maintained that cold‐only stratification does not significantly promote germination even after 4 years of treatment, though the seeds remain viable. Suszka (1985) also recommends fluctuating temperatures during the prechilling warm period as essential for high subsequent germination. Such treatment appears to simulate the natural cycle of seeds in the soil passing through summer and winter, thus explaining why most seeds germinate in the second year.

### 4.2 Ecological Limitations to Seed Production

Seed production by trees is impacted by various environmental factors. Optimal temperature for seed production lies within a specific range, and deviations from this range can negatively affect both quantity and quality. Adequate water availability is critical for seed production, while excess or deficit can hinder it. The amount of light received affects seed production, with overcast conditions or low light decreasing production and high light causing increased seed drop, implying the role of crown and canopy architecture in seed production. Altitude also influences seed production, with temperature and light affecting production differently at different elevations. Seed number, viewed as a functional trait underlying regeneration strategies and population dynamics, is considered a highly variable trait and is very sensitive to environmental conditions, climatic factors, and seed predation (Huang et al. 2021). The number of seeds produced by plant species can vary depending on life stages and environmental conditions. Studies have reasoned that morphological feature of plants, such as biomass and plant size, can determine the average number of seeds produced by plants (Escarre and Thompson 1991; Sakai 1995). Moreover, seed production may show cyclic patterns, giving rise to years of greater seed production in some tree species, which is an efficient reproductive strategy affecting the seed‐to‐seedling transition of plant species (Bogdziewicz et al. 2020; Zhang et al. 2021; Huang et al. 2021).

### 4.3 Is Seed Production and Seed‐to‐Seedling Transition a Limiting Factor?

The seed production observed in the studied stand was sufficient to promote successful regeneration (Figure 1). Over a 4‐year period, 40 trees were monitored annually with a sample size of 10 trees per year to assess whether seed production served as a limiting factor. Studies have suggested that during years of high seed production, seed predation is decreased due to predator satiation from the abundance of food, resulting in improved recruitment and regeneration (Bogdziewicz et al. 2018; Linhart et al. 2014; Wang and Ives 2017; Huang et al. 2021). However, it has also been noted that years of greater seed production may lead to density‐dependent mortality due to the simultaneous emergence of numerous seedlings (Huang et al. 2021). Our observations did not align with previous studies on other tree species, as we did not observe a high density of seedlings following years of greater seed production (Figure S4). However, we do not know why there was poor regeneration of yew. Seeds of *T. baccata* are desiccation‐tolerant (Hong et al. 1998 from Kew); thus, drying of seeds does not explain low regeneration from seeds.

### 4.4 Does Seed Predation Exist in Yew Stand in Hyrcanian Forest?

Our findings, as depicted in informative photographs, suggest that yew seeds may be attractive to animals. This is evidenced by our observations of chewed arils and the presence of seed remains in fecal deposits. This observational evidence could inform future experiments aimed at improving our understanding of yew regeneration in natural ecosystems. Previous studies have established that birds, particularly thrushes, are the main seed dispersers of yew (Farjon 2013). Our observations align with this finding, as the red fleshy aril surrounding the seed (Figure 5a,b) is a key attractant for frugivores and granivores (Levey, Silva, and Galetti 2002; Dennis 2007; Cousens, Dytham, and Law 2008; McConkey et al. 2012). Although we found evidence of yew seed consumption in fecal deposits, we were unable to identify the responsible animal due to the difficulties of monitoring large areas (Figure 5c). Our results also suggest that even small‐sized birds or mammals, such as crows, squirrels, and thrushes, may consume yew seeds, as evidenced by the chewed aril in fecal deposits (Figure 5d).

Seed predation can have both positive and negative effects on yew populations, depending on the level of predation and the availability of alternative food sources for seed predators. On one hand, seed predation can help regulate yew populations by reducing seedling establishment and preventing overpopulation. On the other hand, high levels of seed predation can limit the growth and reproduction of yew populations and reduce the success of forest regeneration efforts.

A significant threat to *T. baccata* regeneration is posed by forest animals, mainly European roe deer (*Capreolus capreolus*), but also red deer (*Cervus elaphus*) and hare (*Lepus europaeus*), which readily gnaw on *T. baccata* regeneration and, in the case of red deer and roe deer, may also defecate in the undergrowth (Mysterud and Østbye 1995; Perrin, Kelly, and Mitchell 2006; Holmes et al. 2009; Widlak 2011). This phenomenon has been observed for years in many *T. baccata* sites and in different geographical regions (Mysterud and Østbye 2004; Weaver and Brown 2004). Preventing the destruction of *T. baccata* regeneration and undergrowth by ungulates can be an important element in the conservation of this species and can have a decisive impact on its survival (Bodziarczyk and Rużyło 2007; Cyzman et al. 2012). Rodents can also pose a threat to new *T. baccata* plantations by effectively destroying the seeds (Hulme 1996, 1997). These species contribute to the removal of *T. baccata* seeds, which sometimes remain dormant in the soil for several years, allowing the seeds to break dormancy and germinate (Hulme 1998; García, Obeso, and Martínez 2005).

### 4.5 Is Seed Dormancy the Reason for Complex Germination Process and Subsequent Poor Regeneration?

Seed dormancy plays a crucial role in regulating the timing of germination in plants, ensuring that germination occurs under conditions favorable for plant establishment. This innate mechanism can delay germination for several seasons, leading to the formation of a seed bank and a high percentage of seed mortality due to seed predation (Baskin and Baskin 2014).

The results of the experiment indicated that the seeds of yew have deep simple morphophysiological dormancy. These findings further elaborate on the nature of dormancy in seeds and its relationship with morphological features. The results suggest that there may be a physiological barrier inhibiting germination in yew seeds. The experiment found that after 3 months of incubation at different temperatures, the seeds were destroyed by fungal infection, preventing us from continuing the germination trials. This result highlights the importance of temperature patterns in the regeneration of yew in natural ecosystems. The results suggest that changes in temperature patterns due to global warming may have disrupted the thermal requirements for dormancy breakage, making it too short to be efficient. Additionally, the results suggest that changes in the sensitivity of seeds to environmental cues in response to changing environments may be an alternative strategy for survival.

We conducted a 3‐year study on the germination of yew from natural seed banks but did not observe any germination from the natural soil seed bank. In these experiments, we sampled forest soil at various depths (5–15 cm) and conducted seedling emergence trials under optimal conditions, though no seedling emergence was observed. Additionally, the number of seeds in the soil was minimal, possibly due to the steep locations from which we collected samples. The study also found that the seeds were viable, but conditions were not suitable for embryo growth. The findings indicate that a complex set of conditions is required for healthy germination and dormancy loss of yew seeds. These findings are consistent with previous research by Heit (1969), which showed that yew seeds germinated to the highest percentage over a 3‐year period, including both warm and cold stratification cycles, which may not be met in the study area due to ecological and environmental factors.

The data shown in Figure S3a,b suggest that changes in climatic factors, particularly in cold seasons, may decrease the chance of yew seeds undergoing the required cycle of warm‐cold stratification for dormancy release. However, previous studies have suggested that the stratification requirements for dormancy loss may be cumulative and can be achieved through more than one cold season (Baskin and Baskin 2014; Maleki et al. 2022). The hypothesis is that seed predation during harsh winter seasons might be affecting the poor regeneration of yew in the Hyrcanian Forest. This is due to the seeds being eaten by animals looking for food while they are waiting for consecutive years to sense the chilling temperature required for cold stratification. Thus, the poor regeneration of yew in the Hyrcanian Forest may be a result of a combination of seed predation and seed dormancy.

Moreover, by characterizing dormancy in seeds, ecologists can gain a better understanding of the factors that regulate germination and the impact of these factors on seedling establishment and forest regeneration. The implications of dormancy characterization for germination and forest regeneration can include identifying strategies to improve germination rates, promoting seedling establishment, and enhancing the success of forest regeneration efforts. We also speculate that herbivory may affect the regeneration of yew from seeds, suggesting that even if a small proportion of seeds are able to germinate and reach the seedling emergence stage, some animals may eat the emerged seedlings.

## 5 Conclusion

This study provides new insights into the potential interplay of factors affecting the regeneration of *T. baccata* in its natural habitat, given the concerns about potential causes of poor regeneration of yew in the natural ecosystem. While years of high seed production were observed, this did not correlate with increased regeneration, suggesting that seed production alone is insufficient to overcome the barriers to yew regeneration. Morphophysiological dormancy and potential seed predation were highlighted as significant factors. The high tolerance of yew seeds to desiccation and the lack of physical barriers to germination suggest that seed predation might be a major obstacle to successful regeneration. Observational evidence of seed predation, such as chewed arils and seed remains in fecal deposits, supports the conclusion that frugivores and granivores play a crucial role in limiting yew seed survival. Despite previous studies linking high seed production with improved regeneration, our observations did not align with these findings, indicating that a more complex set of factors, including seed dormancy and predation, is at play.

Future research should focus on quantifying seed predation and investigating the fate of seedlings to better understand the regeneration dynamics of yew. Understanding these ecological interactions will be vital for developing effective conservation strategies to promote the regeneration and sustainability of yew populations in their natural ecosystems.

## Author Contributions

**Keyvan Maleki:** conceptualization (equal), data curation (equal), formal analysis (equal), funding acquisition (equal), investigation (equal), methodology (equal), resources (equal), software (equal), supervision (equal), validation (equal), visualization (equal), writing – original draft (equal), writing – review and editing (equal). **Paweł Chmielarz:** conceptualization (equal), data curation (equal), formal analysis (equal), funding acquisition (equal), investigation (equal), methodology (equal), project administration (equal), resources (equal), supervision (equal), validation (equal), visualization (equal), writing – review and editing (equal). **Mikołaj Krzysztof Wawrzyniak:** data curation (equal), formal analysis (equal), resources (equal), software (equal), validation (equal), visualization (equal), writing – review and editing (equal). **Kourosh Maleki:** data curation (equal), methodology (equal), resources (equal), software (equal). **Ahmad Maleki:** funding acquisition (equal), investigation (equal), methodology (equal). **Elias Soltani:** data curation (equal), formal analysis (equal), investigation (equal), project administration (equal), supervision (equal), writing – original draft (equal), writing – review and editing (equal).

## Conflicts of Interest

The authors declare no conflicts of interest.

## Supporting information


Data S1.

## Data Availability

The data supporting the findings of this study are included within the manuscript and supplementary file.
