# Supplementary material for: Seed Regeneration in Taxus baccata: Unveiling Ecological Restrictions and Paving the Way for Future Studies
Source: Ecol Evol. 2024 Nov 11;14(11):e70534. doi: 10.1002/ece3.70534 (PMC11554373; doi:10.1002/ece3.70534)
Supplement: Supplementary file 1 — Data S1. [file ECE3-14-e70534-s001.docx]

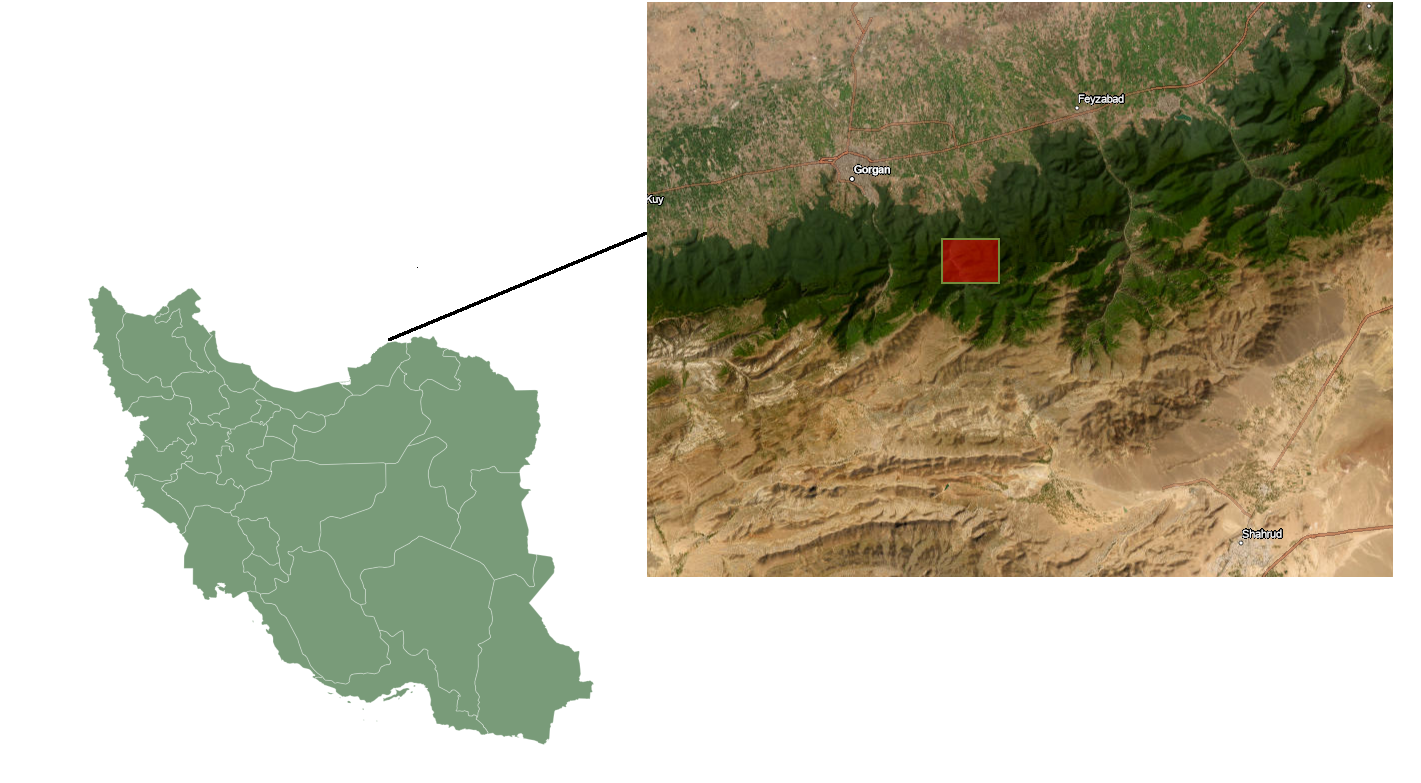


**Figure S1.** A map showing the location of study area in north of Iran.

**Figure S2**. Time-course for water imbibition

**Figure S3.** Monthly temperature and precipitation of past and current climatic condition during growth of *T. baccata* (b,c). Past weather data show a 30-year average, while current data refer to ongoing environmental conditions.

**
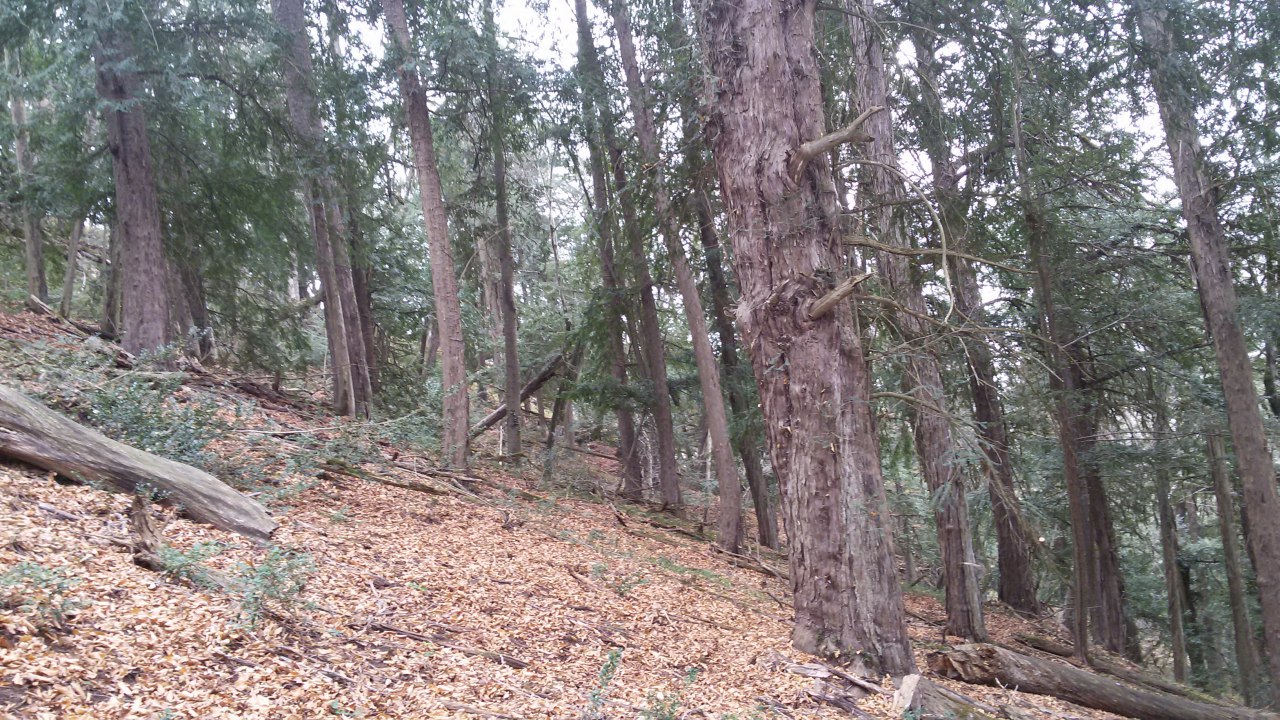
**

**Figure S4.** A photo showing no seedling exist on the surface of understory.

**Table 1S.** Characteristics of trees measured in this study.

| No | Year | Diameter (cm) | crown width (m) | crown (m) | Stalk (stem) (m) | canopy circumference (m) | Number of fruit | Number of seed | Number of branch | Tree height (m) |
| --- | --- | --- | --- | --- | --- | --- | --- | --- | --- | --- |
| 1 | 2016 | 46 | 7.3 | 7.3 | 3.7 | 32.656 | 1888 | 3776 | 24 | 11 |
| 2 | 2016 | 26 | 5.7 | 6.4 | 3.2 | 34.1004 | 2800 | 5600 | 7 | 9.6 |
| 3 | 2016 | 28 | 4.775 | 5 | 2.5 | 18.84 | 2200 | 4400 | 5 | 7.5 |
| 4 | 2016 | 34 | 7.2 | 7 | 3.5 | 30.144 | 2900 | 5800 | 14 | 10.6 |
| 5 | 2016 | 22 | 6.45 | 6.5 | 3.3 | 32.028 | 2634 | 5268 | 7 | 9.8 |
| 6 | 2016 | 37 | 6.6 | 5 | 2.5 | 25.748 | 1285 | 2570 | 9 | 7.5 |
| 7 | 2016 | 46 | 7.5 | 7.8 | 3.9 | 25.12 | 930 | 1860 | 13 | 11.8 |
| 8 | 2016 | 35 | 8.1 | 4.8 | 2.4 | 27.632 | 4320 | 8640 | 8 | 7.2 |
| 9 | 2016 | 33 | 7.8 | 7 | 3.5 | 37.68 | 1253 | 2506 | 35 | 10.5 |
| 10 | 2016 | 38 | 6.65 | 5.4 | 2.7 | 32.028 | 1870 | 3740 | 12 | 8.1 |
| 1 | 2017 | 46 | 7.8 | 6.2 | 3.1 | 30.144 | 2236 | 4472 | 7 | 9.3 |
| 2 | 2017 | 28 | 7.6 | 5 | 2.5 | 26.376 | 1693 | 3386 | 13 | 7.5 |
| 3 | 2017 | 29 | 7.2 | 5.7 | 2.9 | 33.284 | 1498 | 2996 | 16 | 8.6 |
| 4 | 2017 | 31 | 6.45 | 6.2 | 3.1 | 38.308 | 3026 | 6052 | 21 | 9.3 |
| 5 | 2017 | 37 | 6.2 | 7.2 | 3.6 | 37.052 | 2560 | 5120 | 9 | 10.9 |
| 6 | 2017 | 41 | 7.65 | 7.7 | 3.9 | 28.888 | 3874 | 7748 | 6 | 11.6 |
| 7 | 2017 | 46 | 7.8 | 5.2 | 2.6 | 31.4 | 3117 | 6234 | 17 | 7.9 |
| 8 | 2017 | 25 | 7.5 | 5.6 | 2.8 | 33.284 | 2469 | 4938 | 14 | 8.5 |
| 9 | 2017 | 32 | 6.65 | 4.7 | 2.4 | 37.052 | 1594 | 3188 | 11 | 7.1 |
| 10 | 2017 | 30 | 6.1 | 6.4 | 3.2 | 30.772 | 3872 | 7744 | 16 | 9.6 |
| 1 | 2018 | 26 | 5.7 | 7.1 | 3.6 | 29.516 | 1621 | 3242 | 21 | 10.7 |
| 2 | 2018 | 76 | 7.85 | 5.4 | 2.7 | 28.888 | 1346 | 2692 | 16 | 8.2 |
| 3 | 2018 | 51 | 6.75 | 6.4 | 3.2 | 32.028 | 2664 | 5328 | 7 | 9.6 |
| 4 | 2018 | 39 | 6.35 | 6 | 3.0 | 38.936 | 2436 | 4872 | 22 | 9.1 |
| 5 | 2018 | 44 | 5.1 | 5.7 | 2.9 | 35.796 | 3411 | 6822 | 24 | 8.6 |
| 6 | 2018 | 37 | 7.9 | 5.6 | 2.8 | 37.052 | 1483 | 2966 | 14 | 8.4 |
| 7 | 2018 | 46 | 6.85 | 5 | 2.5 | 24.492 | 2269 | 4538 | 11 | 7.6 |
| 8 | 2018 | 52 | 5.7 | 4.9 | 2.5 | 35.168 | 2354 | 4708 | 10 | 7.4 |
| 9 | 2018 | 48 | 7.15 | 4.6 | 2.3 | 24.492 | 4156 | 8312 | 16 | 7 |
| 10 | 2018 | 31 | 7.5 | 5.7 | 2.9 | 30.144 | 2964 | 5928 | 8 | 8.6 |
| 1 | 2019 | 37 | 7.5 | 6 | 3.0 | 35.168 | 2364 | 4728 | 23 | 9.1 |
| 2 | 2019 | 39 | 6 | 6.2 | 3.1 | 37.052 | 1339 | 2678 | 22 | 9.4 |
| 3 | 2019 | 42 | 5.55 | 6.8 | 3.4 | 21.98 | 4201 | 8402 | 24 | 10.3 |
| 4 | 2019 | 24 | 6.25 | 7.4 | 3.7 | 39.564 | 1863 | 3726 | 16 | 11.1 |
| 5 | 2019 | 49 | 5.65 | 4.8 | 2.4 | 35.168 | 1660 | 3320 | 10 | 7.2 |
| 6 | 2019 | 28 | 6.95 | 4.8 | 2.4 | 20.096 | 2100 | 4200 | 7 | 7.3 |
| 7 | 2019 | 33 | 7.4 | 4.6 | 2.3 | 31.4 | 2306 | 4612 | 5 | 6.9 |
| 8 | 2019 | 37 | 7.45 | 5.5 | 2.8 | 38.308 | 1421 | 2842 | 9 | 8.3 |
| 9 | 2019 | 42 | 7.1 | 6 | 3.0 | 37.052 | 3096 | 6192 | 13 | 9.1 |
| 10 | 2019 | 46 | 6.6 | 5.8 | 2.9 | 33.284 | 2596 | 5192 | 17 | 8.8 |
